# Graphic type differentiation of cell count data for diagnosis of early and late periprosthetic joint infection: A new method

**DOI:** 10.3233/THC-231006

**Published:** 2024-09-03

**Authors:** Bernd Fink, Marius Hoyka, Benedikt Paul Blersch, Hannsjörg Baum, Florian Hubert Sax

**Affiliations:** a Department for Joint Replacement, Rheumatoid and General Orthopaedics, Orthopaedic Clinic Markgröningen, Markgröningen, Germany; b Orthopaedic Department, University Hospital Hamburg-Eppendorf, Hamburg, Germany; c Institute for Laboratory Medicine and Transfusion Medicine, RKH Regionale Kliniken Holding und Services GmbH, Ludwigsburg, Germany

**Keywords:** Leucocyte count, synovia, periprosthetic joint infection, cell counter

## Abstract

**BACKGROUND:**

Graphic type differentiation of cell count data of synovial aspirates is a new method for the diagnosis of early and late periprosthetic joint infection.

**OBJECTIVE:**

The aim of the study was to analyse if the same 6 LMNE-types can be differentiated in the new Yumizen H500 cell counter as it was the case for the old cell counter ABX Pentra XL 80 of previous publications, to verify if the erythrocyte and thrombocyte curves of the new device give additional information and to calculate the difference of cell count in LMNE-type I and III (with abrasion) in the cell counter and in the manual counting chamber (Neubauer improved).

**METHODS:**

450 aspirates of 152 total hip arthroplasties and 298 knee arthroplasties obtained for the diagnosis of periprosthetic joint infection were analysed with the Yumizen H500.

**RESULTS:**

All LMNE-matrices of the 450 aspirates could assigned to one of the six LMNE-types. There were 76 LMNE-type I, 72 LMNE-type II, 14 LMNE-type III, 241 LMNE-type IV, 36 LMNE-type V and 12 LMNE-type VI. The erythrocyte and thrombocyte distribution curves were very helpful for differentiation of hematoma and infection. The cell count in the manual counting procedure was lower than in the cell counter: for the LMNE-type I (abrasion type) the median of the difference was 925/μL (median) and for the LMNE-type III (combined type of infection and abrasion) 3570/μL (median).

**CONCLUSION:**

The described graphic type differentiation is a new and helpful method for differentiation of hematoma and early PJI as well as abrasion and late PJI.

## 1. Introduction

Periprosthetic joint infection (PJI) is a devastating complication of arthroplasty procedures and has many consequences. The level of incidence for primary total hip and knee arthroplasties ranges between 1% and 2% on average [1, 2, 3, 4]. With regard to revision arthroplasty, it is even higher and ranges between 2% and 7.5% [5, 6, 7]. The accuracy of the preoperative diagnosis of possible PJI is critical because, if it remains unrecognized before or during revision, it leads to a high risk of failure due to recurrence of the infection [8, 9].

There is no single diagnostic test with an accuracy of 100% to verify or rule out an early/acute or late/chronic periprosthetic joint infection; thus, combinations of different diagnostic tests are helpful [10, 11, 12, 13]. One important diagnostic method for PJI is the determination of the leukocyte count (WBC) in the joint synovia. Some authors consider it one of the most important diagnostic parameters [14, 15, 16] and it is included as one of the minor criteria in the definition of the PJI, the MSIS criteria and the more recent ICM criteria [10, 17, 18].

To set the cut-off value of WBC for early/acute and late/chronic periprosthetic joint infection is difficult for several reasons and therefore varies between the different published reports. In a consensus meeting, the threshold of WBC in the aspirate for acute periprosthetic infection (within 4 weeks after implantation or onset of symptoms in acute hematogenous infections) was set at 10,000 cells/μL, because different publications quoted threshold values between 8,910 cells/μL und 12,800 cells/μL [19, 20, 21, 22, 23, 24]. However, this value corresponds to leukocyte numbers that may also be present in normal blood, so that postoperative hematomas in the joint may also exhibit leukocyte counts at this level [19, 21]. Another important reported parameter is the percentage of polymorphonuclear leukocytes in the aspirate [10, 17, 18, 23], whereby the cut-off value was determined to be 89% in several studies [19, 20, 21] and 79.5% in another study [24]. The International Consensus Group meeting fixed the cut-off value at 90% PMN in the aspirate [23]. However, the percentage of polymorphonuclear leukocytes in the aspirate can also be influenced by the presence of a hematoma. Because postoperative edema and swelling can make it difficult to distinguish clinically between early periprosthetic infection and hematoma, additional tools or parameters for the differentiation of these two situations would be helpful.

In chronic/late periprosthetic joint infections, the variance of thresholds for leukocyte number in the synovial aspirate is even higher [25]. Thresholds between 1,100 cells/μL [26] and 5,000 cells/μL [27] are described in the literature [16, 25]. This leads to the setting of the threshold at > 3,000 cells/μL (where PJI is confirmed) [23, 28], and < 1,500 cells/μL (where PJI is unlikely) [28]. Factors that lead to this broad variance of thresholds include the time after the operation, the duration of the symptoms, the detected microorganisms, administered antibiotics, and the authors only setting the cut-off value after receiving the results, depending on the prevalence of high sensitivity or specificity [29].

In a previous paper, we could show that this variance was also caused by abraded wear particles from the articulating surface of the artificial joint (polyethylene particles or metal particles) that were detected and counted by the cell counter in addition to the leucocytes in the aspirate [25]. We could differentiate between leucocytes and wear particles in the aspirate because of the different behaviour with regard to light absorption and we could also identify different graphical types (so called LMNE-types) of the aspirate: type I (abrasion type), type II (infection type), type III (mixed type of infection and abrasion), type IV (indifference type) [25]. We could show that these graphical LMNE types had a significantly high correlation to the histological types I–IV (with the same names) according to Krenn et al. [30, 31] and Morawietz [32].

In a second paper, we expanded the number of graphical LMNE-types to include the differentiation of periprosthetic infection and hematoma: type V (hematoma) and type VI (mixed type of infection and hematoma) [33]. Differentiation of LMNE types into infection (types II and VI) and non-infection (types IV and V) resulted in a sensitivity of 100%, a specificity of 97.3%, and a positive likelihood ratio of 37.0 [33].

**Figure 1.**
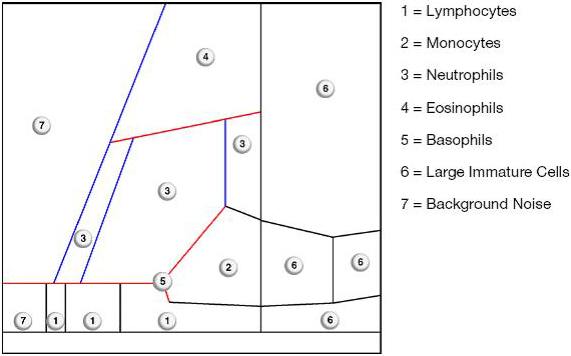
a: LMNE matrix with the different areas corresponding to the leukocyte populations and the NOISE area of the ABX Pentra XL 80. b: LMNE matrix with the different areas corresponding to the leukocyte populations and the NOISE area of the Yumizen H500.

The cell counter ABX Pentra XL 80 (Horiba Medical, Montpellier, France) was used for the investigations described in those two papers. In the meantime, the next generation of these cell counters is on the market – the Yumizen H500 (Horiba Medical, Montpellier, France) – and has replaced the old ones. As a result, the LMNE-plots look somewhat different to those of the ABX Pentra XL 80 (Fig. 1a and b). 

**Figure:**
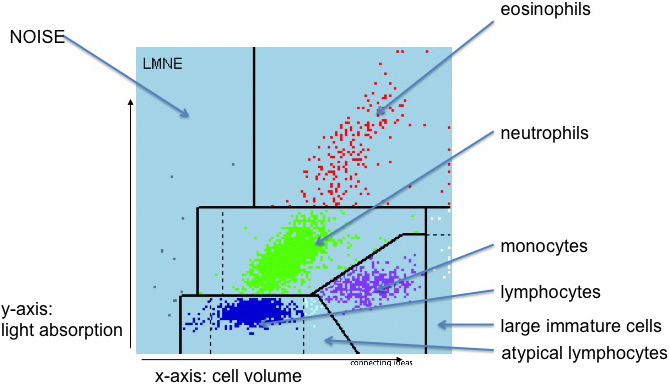

Therefore, the questions of the current study were:

1. Can the same 6 LMNE-types be differentiated in the new Yumizen H500 as was the case for the old ABX Pentra XL 80?
2. Does the new cell counter give additional information for the differentiation of these 6 types?
3. What difference of cell count are measured in LMNE-type I and III (with abrasion) in the cell counter and in the manual counting chamber (Neubauer improved).

## 2. Material and methods

450 aspirates of 152 total hip arthroplasties and 298 knee arthroplasties obtained for the diagnosis of periprosthetic joint infection were analysed with the Yumizen H500. There were 239 female and 211

**Figure 2.**
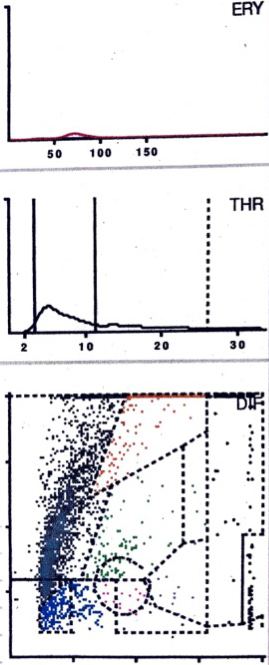
LMNE matrix of a type I with metal debris particles of a 84-year old female patient with knee arthroplasty. There is a cluster in the NOISE-area. The measured “cell count” was 1970 cells/μL and the percentage of polymorphnuclear neutrophil leukocytes (PMN) was 23.4%.

**Figure 3.**
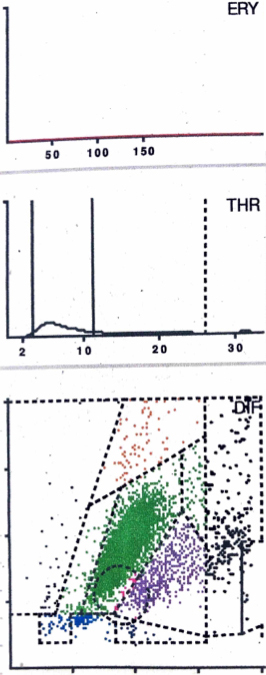
LMNE matrix of a type II (infection type) with a cluster of data points in the area of the neutrophil leukocytes and no peak in the erythrocyte field in a 57-year old patient with a late periprosthetic joint infection of a total knee arthroplasty. The measured cell count was 9840 cells/μL and 89.4% PMN.

male patients with a mean age of 70.5 ± 16.8 (range 50–90) years. Systemic inflammatory diseases like rheumatoid arthritis were excluded, because these diseases can have leukozytes in the joint beside a PJI [34]. The Yumizen H500 (Horiba Medical, Montpellier, France) is a laboratory diagnostic device for the analysis of the cell count and the WBC-differentiation of blood and also of other body fluids. Here we selected the so-called 5-DIFF mode from the various processing modes available. A total of 26 laboratory parameters are recorded, including the 5 cell types – eosinophils, neutrophils, monocytes, lymphocytes and basophils – as well as atypical lymphocytes and large, immature cells. These cell types are graphically mapped in a so-called LMNE matrix depending on their cell volume (x-axis) and their light scattering or refraction and absorption (y-axis) (Fig. 1b). The analysis is based on an impedance measurement, flow cytometry and cytochemistry. The volume difference of the cells due to the impedance measurement is shown graphically on the X-axis and the differentiation of the light absorption in flow cytometry on the Y-axis of the LMNE matrix. This enables the graphical assignment and thus differentiation of the 4 leukocyte populations: lymphocytes, monocytes, neutrophils and eosinophils (Fig. 1b). Impurities appear in the so-called NOISE area of the LMNE matrix; in our synovial analyses of joints with prostheses these are abraded wear particles (Fig. 1b). The LMNE-matrices of each patient were allocated to one of the different LMNE-types (I to VI) (if possible). Additionally, the cell count and percentage of polymorphonuclear leucocytes were also measured by the Yumizen H500 cell counter. When a LMNE-type with abrasion (types I and III) was identified, the cell count was also determined manually using a Neubauer improved counter chamber (Carl Roth GmbH, Karlsruhe, Germany). Additionally the cell counter Yuminzen H500 gave curves for the erythrocyte and thrombocyte distribution of the aspirate with the size of the cell on the x-axis and the amount of cells on the y-axis (Figs 2–7).

The evaluations and assignment of the matrices to the different types were carried out twice by 2 examiners (BF and MH) independently of one another. IBM SPSS Version 24 (IBM Corp., Aemonk, NY, USA) and Microsoft Excel (Microsoft, Redmond, WA, USA) were used for statistical analysis. Categorical variables are depicted as frequencies, while continuous variables are shown as medians, standard deviation and ranges. 

## 3. Results

All LMNE-matrices of the 450 aspirates could assigned to one of the six LMNE-types. There were 76 LMNE-type I, 72 LMNE-type II, 14 LMNE-type III, 241 LMNE-type IV, 36 LMNE-type V and 12 LMNE-type VI (Table 1). The assignment by the two examiners (BF, MH) showed a high reliability, with an intrarater intraclass correlation coefficient of 0.99 and of 0.98 between raters, respectively.

**Table 1:** Assignment of the aspirates to the different LMNE-types and additional identification of abrasion debris (substantial in type I and III and slight in the other types) as well as hematoma (substantial in type V and VI and slight in the other types)

LMNE-type	N	With abrasion	With hematoma
I (abrasion type)	76	76	3
II (infection type)	72	9	20
III (mixed type = infection + abrasion)	14	14	2
IV (indifference type)	241	88	37
V (hematoma type)	36	7	36
VI (mixed type = infection + hematoma)	12	3	12

**Figure 4.**
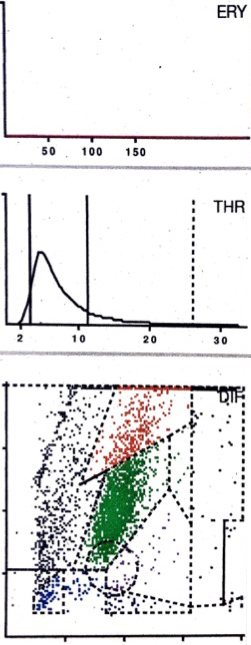
LMNE matrix of a type III (combined type of infection and abrasion) with a cluster in the area of the neutrophil leukocytes and a second cluster in the NOISE area as well as no peak in the erythrocyte field in a 83-year old female patient with a periprosthetic joint infection of a hip arthroplasty. The measured cell count was 2940 cells/μL with 79.6% PMN.

For the detection of hematoma, the erythrocyte and thrombocyte distribution curves were very helpful in indicating the existence of a significant number of erythrocytes and thrombocytes. This allowed the differentiation between the hematoma (type V) with a high number of leucocytes, and infection (type II) to be carried out more easily: the hematoma exhibited a peak in the erythrocyte and thrombocyte curve whereas the infection type did not (Figs 3 and 6). The higher the peak of the erythrocyte and thrombocyte curve, the more erythrocytes and thrombocytes (and more hematoma) were in the aspirate.

**Table 2:** Cell counts and percentages of polymorphonuclear leucocytes of the different types

LMNE-type	Cell count (/μL) (median; min–max)	% PMN (median; min–max)
Type I	1,195; 274–158,070	24.6; 4.0–59.2
Type II	6,595; 1,130–179,510	81.2; 69.1–94.1
Type III	9,105; 1,620–93,290	69.5; 39.3–89.8
Type IV	560; 90–3,120	31.8; 3.7–77.9
Type V	2,740; 220–12,980	58.2; 20.6–89.0
Type VI	56,880; 6,380–287,630	80.8; 52.9–91.5

**Figure 5.**
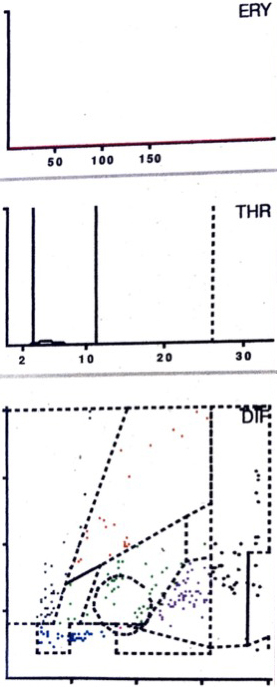
LMNE matrix of a type IV (indeterminate type) with no obvious data point clusters or increase in cell types or particles in a 62-year old female patient. The measured cell count was 400 cells/μL with 22.5% PMN.

**Figure 6.**
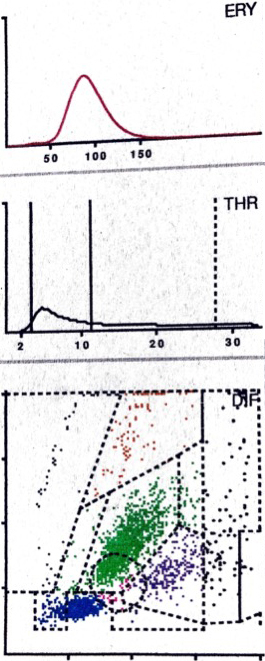
LMNE matrix of a type V (hematoma) 75-year old female patient with a hematoma 6 weeks after knee arthroplasty. There is a cluster in the field of the lymphocytes and another clear cluster in the fleld of the neutrophils and a peak in the erythrocyte field. The measured cell count” was 10,120 cells/μL with 59.2% PMN.

**Figure 7.**
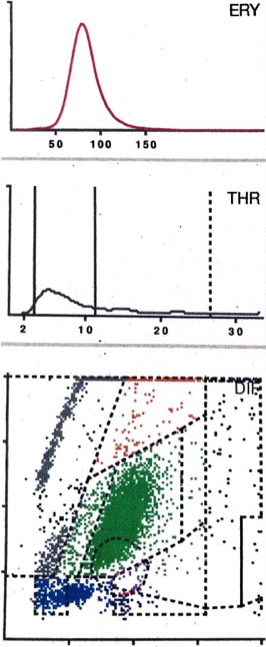
LMNE matrix of a type VI (combined type II of hematoma and infection) 5 weeks after implantation of a hip arthroplasty with a cluster in the area of the neutrophil leukocytes and increases in the other areas of the white blood cells as well as a peak in the erythrocyte field in a 72 old female patient with an early periprosthetic joint infection. The measured cell count was 15200 cells/μL with 84.4% PMN.

The cell counts and percentages of polymorphonuclear leucocytes of the different types are shown in Table 2. The cell count in the manual counting procedure was lower than in the cell counter: for the LMNE-type 1 (abrasion type) the median of the difference was 925/μL with a range of 90 to 45,370 cells/μL and for the LMNE-type III (combined type of infection and abrasion) 3570/μL (median) with a range of 1,200 to 10,210 cells/μL, respectively.

## 4. Discussion

The analysis of the aspirates by differentiating the LMNE-matrices into the LMNE-types I to VI with the ABX Pentra XL 80 (Horiba Medical, Montpellier, France), as described in the two previous publications, could clearly be reproduced with the new generation of this laboratory diagnostic device, the Yumizen H500 (Horiba Medical, Montpellier, France) [25, 33]. Therefore, it can be expected that analysing joint aspirates with this new device will give additional information for the diagnostic of periprosthetic joint infections, as was the case for the older device. 

On the basis of the results of an earlier paper, in late/chronic PJI, the additional use of the LMNE-type classification can help to distinguish between a true infection type and a debris type, which would otherwise lead to concluding an incorrectly high cell number [25]. The much lower number of cells counted in the manual counting chamber support the hypothesis that abraded wear particles can be mis-counted as leucocytes in the cell counter, especially in LMNE-type I and III aspirates. Above all, this method can be used to differentiate leukocytes from metallic abrasion particles, which appears to be of particular importance, since joint aspirates with metal abrasion can look like pus, can have very high cell counts, elevated CRP- and alpha-defensin values and can thus wrongly be interpreted as a periprosthetic infection [35, 36, 37, 38, 39, 40]. Furthermore, the combination of cell count-analysis and LMNE types II or III-determination enabled us in the first paper to reduce the cut-off value for late PJI of the cell count in the aspirate to 1,400 cells/μL without losing high sensitivity (sensitivity of 90.2% and a specificity of 91.9%, receiver operation characteristics curve) [25]. The combination of cell counting and the graphic representation in the LMNE matrix means that fewer periprosthetic infections are overlooked and the diagnostic value of the cell count analysis in the joint aspirate is increased. 

Based on the results of another previous paper, the LMNE matrix evaluation during the first weeks after implantation of an endoprosthesis can help to differentiate infections from hematomas, especially in the cases with cell counts in the borderline range of the threshold value chosen by the ICM. This is because cluster plots in the LMNE-fields representing lymphocytes, basophils and/or eosinophils show that a significant part of the cluster in the neutrophil leukocyte field must be due to a hematoma. Therefore, cell count values in the borderline range can be corrected downward and thus not be interpreted as an infection. Hereby, the additional erythrocyte and thrombocyte distribution plot of the Yumizen H500 helps to identify a hematoma and distinguish it from an infection. Therefore, this method can be used to differentiate infections from hematoma. This is of particular importance because postoperative edema and swelling can make it difficult to distinguish these two situations clinically and the cell count in a hemarthrosis can be at the level of the threshold for infection described by the ICM and other authors [19, 20, 21, 22, 23, 24].

This study has some limitations. First of all, only the reproducibility of the differentiation of the 6 LMNE-types with the new laboratory device was analyzed. The accuracy, sensitivity and specificity of the LMNE-type-differentiation with the Yumizen H500 have to be analyzed in further studies using criteria like that of the MSIS for comparison. Moreover, the type classification is somewhat dependent on the personal interpretation and experience of the examiner. Even if the reliability of this type classification was very high in our study, this does not rule out a certain subjectivity in the interpretation of the LMNE matrices.

## 5. Conclusion

The graphic representation of the cell count analysis of synovial aspirates of joints with endoprostheses is a new and helpful method for determining the cell count of the aspirate and to differentiate between true early periprosthetic infections with increased leukocyte count and hematomas in the early postoperative period. In addition, the ability to differentiate real late PJIs with increased leukocyte counts and apparently increased numbers due to particle abrasion increases the diagnostic value of the cell count analysis in such cases. In our opinion, it should therefore be included in the diagnostic armamentarium used to identify periprosthetic joint infections in future.
